# Ustekinumab-Induced Fatal Acute Heart Failure in a Young Female: A Case Report

**DOI:** 10.14797/mdcvj.1076

**Published:** 2022-07-12

**Authors:** Mahmoud Abdelnabi, Saif ElNawaa, Juthipong Benjanuwattra, Mohamed Elmassry, Nandini Nair

**Affiliations:** 1 Texas Tech University Health Sciences Center, Lubbock, Texas, US; 2 Medical Research Institute, Alexandria University, Alexandria, Egypt

**Keywords:** acute heart failure, ustekinumab, pityriasis rubra pilaris, biological agents

## Abstract

Data regarding short- and long-term cardiovascular complications of biological agents is lacking. Herein, the authors reported a case of abrupt onset of acute heart failure induced by ustekinumab treatment for resistant pityriasis rubra pilaris in a young healthy female with no past cardiac history. Although medical management was immediately initiated for cardiogenic shock, the patient passed away.

## Introduction

Ustekinumab is a monoclonal antibody within the interleukin (IL)-12/23 inhibitors class that is approved by the US Food and Drug Administration (FDA) for moderate to severe plaque psoriasis, active psoriatic arthritis, and inflammatory bowel diseases. Promising results were shown in patients with various dermatological conditions refractory to traditional therapies.^1^ However, there are insufficient data regarding the relation between ustekinumab therapy and heart failure.

## Case report

**Figure 1:**
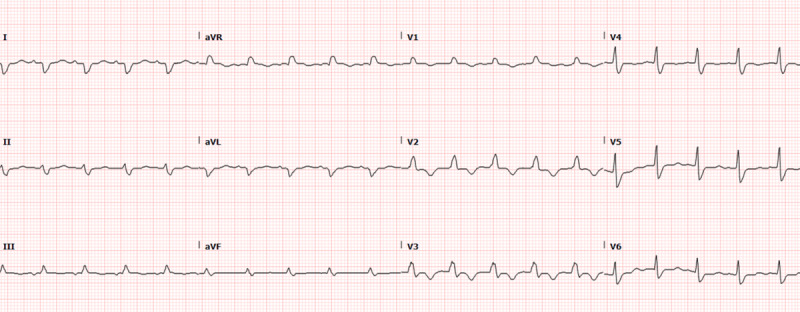
Electrocardiogram showed sinus tachycardia 120 beats/minute, right bundle branch block, and left posterior fascicular block.

A 21-year-old female presented in 2021 with extensive erythematous skin rash that was provisionally diagnosed as severe eczema. She was treated conventionally with antihistamines and corticosteroids, but her condition did not improve. After referral to the dermatology department, she was diagnosed with pityriasis rubra pilaris and started on dupilumab, which was continued for 8 months with no response. She was then started on ustekinumab and reported improvement of her skin lesions. However, within a few days, she started to complain of gradually worsening shortness of breath and bilateral lower extremity edema. She had no previous medical history of cardiovascular risk factors or family history of heart disease. On clinical examination, she was distressed, tachypneic with a respiratory rate of 25, tachycardiac with a heart rate of 120 beats/minute, and with blood pressure of 90/60 mm Hg. Cardiac examination was significant for jugular venous dilatation, pansystolic murmur over the mitral area, and bilateral lower limb edema. Lung auscultation revealed bilateral diffuse crepitations. Her laboratory workup was significant for markedly elevated pro B-type natriuretic peptide (NT-proBNP), renal and liver functions, lactic acidosis, and slightly elevated troponin levels. Electrocardiogram showed sinus tachycardia 120 beats/minute, right bundle branch block, and left posterior fascicular block (Figure 1). Urgent transthoracic echocardiography (Videos 1, 2) revealed severe global hypokinesia with biventricular systolic dysfunction, severe mitral regurgitation, mild tricuspid regurgitation, and multiple echogenic masses in the right ventricular apex that likely were thrombi. She was started immediately on high doses of inotropes, vasopressors, diuretics, and anticoagulation without a significant response. Her family was counseled for transfer to another medical facility where she could receive mechanical circulatory support, but due to her critical condition, they opted to proceed with noninvasive medical management. A few days later, she went into cardiac arrest. Cardiopulmonary resuscitation was performed, but she passed away.

**Video 1:** Transthoracic echocardiology showed severe global hypokinesia with biventricular systolic dysfunction, severe mitral regurgitation, and multiple echogenic masses in the right ventricular apex that likely were thrombi; also at https://youtu.be/51CVBgyW-eg.

**Video 2:** Transthoracic echocardiology showed severe global hypokinesia with biventricular systolic dysfunction, severe mitral regurgitation, and multiple echogenic masses in the right ventricular apex that likely were thrombi; also at https://youtu.be/gpXIdiaF1wk.

## Discussion

This is a case of abrupt onset of acute heart failure induced by ustekinumab treatment for resistant pityriasis rubra pilaris in a patient without any family or medical history of cardiovascular disease. Similar to the case reported by Beroukhim et al.,^2^ reversible ustekinumab-induced heart failure for treatment of plaque-type psoriasis was reported in a 46-year-old female patient with no past cardiac history.

Several biologic agents are available for the treatment of moderate-to-severe psoriasis. Ustekinumab, a human monoclonal antibody targeting the shared p40 subunit of interleukin-12 (IL-12) and interleukin-23 (IL-23), has been recently approved by the FDA for patients with immune-mediated inflammatory diseases, including moderate-to-severe plaque psoriasis and Crohn’s disease.^3^ Data from phase III clinical trials demonstrated promising efficacy and safety profile with rapid improvement in psoriasis area and severity index.^3,4^ Common side effects include upper respiratory tract infections, nasopharyngitis, headache, and arthralgias.^4^

The mechanism and effects of ustekinumab on the heart remain largely unknown. IL-23, which plays a pivotal role in the differentiation of T helper 17 cells (Th17) and subsequent production of interleukin-17 (IL-17), is inhibited by ustekinumab.^5^ The role of IL-17 in atherosclerosis remains controversial.^5^ Several studies demonstrated its proatherogenic effect, whereas a plaque-stabilizing effect was noted in the setting of enhanced IL-17 production with suppressed interferon-γ.^5^ According to the French national health insurance database consisting of 9,290 patients treated with ustekinumab, there was a statistically significant correlation between ustekinumab and severe atherosclerotic cardiovascular events—including acute coronary syndrome and ischemic stroke—within 6 months after initiation in those with high cardiovascular risk.^6^ Also, from a prospective study of 981 patients with acute myocardial infarction, lower serum levels of IL-17 were associated with a higher risk of death and recurrent myocardial infarction.^7^ The observed findings from both studies were likely attributed to the impaired regulatory and atheroprotective role of IL-17. Additionally, data from an in vivo study of myocardial infarction mice showed that IL-23 deficiency was associated with adverse remodeling and myocardial inflammation.^8^

In addition to the atherothrombotic events, twelve cases of in-hospital mortality due to heart failure were reported in the French national health insurance database.^6^ Data from PHOENIX 2—a multicenter, phase III, randomized, placebo-controlled study of 1,212 patients with moderate-to-severe psoriasis—revealed two cases of new-onset heart failure associated with 90 mg ustekinumab after 806 and 1,217 days of treatment, respectively.^9^ Previous evidence regarding the use of ustekinumab and cardiovascular events was well summarized and recently published.^10^ However, most of the studies included either a short follow-up or only focused on a composite of major cardiovascular events that did not involve heart failure. Besides heart failure, venous thrombosis has been reported in 115 patients (0.18%) from a total of 65,635 patients according to a phase IV clinical trial.^11^ The echocardiographic finding of large right ventricular thrombi in our patient also raises concern for this possible adverse event, although it was more likely secondary to heart failure.

Controversial results showing favorable effects to the heart were obtained from a trial of 150 psoriasis patients randomly treated with ustekinumab, etanercept, or cyclosporine. In this trial, IL-12/23 inhibition led to improved left ventricular global longitudinal strain and lower NT-proBNP levels.^12^ IL-12, a major pro-inflammatory cytokine involved in psoriasis, is elevated in the failing heart; therefore, its cardioprotective effect was presumably due to IL-12 inhibition. The detrimental effect of IL-17 on adverse cardiac remodeling was also demonstrated in animal models,^13,14^ so it is plausible that ustekinumab-mediated IL-17 inhibition may contribute to the improved outcomes in this study.

## Conclusion

Given the controversy showing both cardioprotective and detrimental effects secondary to IL-12/23 inhibition, the risks and benefits of initiating ustekinumab, particularly in those with cardiovascular risk factors or established cardiovascular diseases, should be thoroughly assessed until the exact mechanistic relationship can be ascertained. It should be noted that heart failure could also develop in those without prior cardiac history. Although the incidence seems to be low, interval monitoring with echocardiography may be beneficial for early detection of heart failure, thus allowing prompt discontinuation of ustekinumab.
